# Clinical and Quality of Life Benefits for End-Stage Workers’ Compensation Chronic Pain Claimants following H-Wave^®^ Device Stimulation: A Retrospective Observational Study with Mean 2-Year Follow-Up

**DOI:** 10.3390/jcm12031148

**Published:** 2023-02-01

**Authors:** Alan Trinh, Tyler K. Williamson, David Han, Jeffrey E. Hazlewood, Stephen M. Norwood, Ashim Gupta

**Affiliations:** 1 Indiana University School of Medicine, Indianapolis, IN 46202, USA; 2 University of the Incarnate Word School of Osteopathic Medicine, San Antonio, TX 78235, USA; 3 Department of Management Science and Statistics, University of Texas at San Antonio, San Antonio, TX 78249, USA; 4 Jeffrey E. Hazlewood, MD, PC, Physical Medicine and Rehabilitation, Lebanon, TN 37090, USA; 5 Retired Orthopaedic Surgeon, Austin, TX 78738, USA; 6 Future Biologics, Lawrenceville, GA 30043, USA; 7 Regenerative Orthopaedics, Noida 201301, UP, India

**Keywords:** H-Wave^®^, electrotherapy, neurostimulation, quality of life, functional status, pain reduction, chronic pain, opioids, polypharmacy, workers’ compensation

## Abstract

Previously promising short-term H-Wave^®^ device stimulation (HWDS) outcomes prompted this retrospective cohort study of the longer-term effects on legacy workers’ compensation chronic pain claimants. A detailed chart-review of 157 consecutive claimants undergoing a 30-day HWDS trial (single pain management practice) from February 2018 to November 2019 compiled data on pain, restoration of function, quality of life (QoL), and polypharmacy reduction into a summary spreadsheet for an independent statistical analysis. Non-beneficial trials in 64 (40.8%) ended HWDS use, while 19 (12.1%) trial success charts lacked adequate data for assessing critical outcomes. Of the 74 final treatment study group charts, missing data points were removed for a statistical analysis. Pain chronicity was 7.8 years with 21.6 ± 12.2 months mean follow-up. Mean pain reduction was 35%, with 89% reporting functional improvement. Opioid consumption decreased in 48.8% of users and 41.5% completely stopped; polypharmacy decreased in 36.8% and 24.4% stopped. Zero adverse events were reported and those who still worked usually continued working. An overall positive experience occurred in 66.2% (*p* < 0.0001), while longer chronicity portended the risk of trial or treatment failure. Positive outcomes in reducing pain, opioid/polypharmacy, and anxiety/depression, while improving function/QoL, occurred in these challenging chronic pain injury claimants. Level of evidence: III

## 1. Introduction

Chronic pain, defined as pain that persists past normal healing times or for at least 3–6 months, has been estimated to eventually affect up to 20% of the world’s population [1]. Chronic pain often interferes with an individual’s ability to work, placing additional financial burdens on families, government, and insurers [2]. One global impact study concluded that the annual cost of treating chronic pain ranged between USD 560 billion to USD 635 billion [3].

Typical chronic pain management often involves a combination of nonpharmacologic treatments such as physical therapy, cognitive behavioral therapy, and transcutaneous electrical nerve stimulation (TENS), added to pharmacologic agents including nonsteroidal anti-inflammatory drugs (NSAIDs), acetaminophen, topical anesthetics, opioids, and other controlled substances [4]. Opioids, while retaining utility in providing acute pain relief following surgery or trauma, only target the symptoms and fail to address the pain source, being associated with high addiction rates and life-threatening adverse effects with prolonged use [5,6,7]. Chronic pain patients often have other comorbidities, thus placing them at an increased risk for the problematic side effects of opioids [8,9]. Even non-opioid pharmacologic alternatives, which are often prescribed in a multimodal approach (polypharmacy), including but not limited to nonsteroidal, antidepressant, anticonvulsant, cannabinoid, and natriuretic drugs, involve moderate risk, with some being quite expensive. Unfortunately, many chronic pain patients have refractory painful conditions (which may not be amenable to definitive and curable treatment options), and end up spending inordinate time, effort, and money experimenting with various questionable approaches [10]. In light of the current ongoing opioid epidemic and an increasing prevalence of chronic pain, there is a growing need to identify safer, cost-effective multimodal therapeutic regimens that not only mitigate the perception of pain, but also target the pain-generating source [11,12].

H-Wave^®^ device stimulation (HWDS) is a unique type of transcutaneous electrotherapy that uses a specific proprietary waveform (biphasic, exponentially decaying, low frequency, long pulse duration) to stimulate muscle fiber contractions, which are non-fatiguing and low-tension, mimicking natural voluntary motor contractions [13]. This leads to increased blood flow via nitric oxide (NO)-dependent vasodilation, angiogenesis (formation of new blood vessels), resolution of edema, and anesthesia (in high-frequency mode) [13,14,15]. The H-Wave^®^ device utilizes a propriety waveform and parameters that are distinct from other available electrical stimulation devices such as TENS and NMES and should not be confused with the H waves associated with electromyography and the Hoffmann reflex. A recent critical review of the H-Wave^®^ literature found significant benefits for a variety of musculoskeletal and neurological disorders, virtually without side effects, resulting in the lowering of pain medication use in 40–65% of neuropathic pain patients, while invariably improving functional status [13].

In addition to its clinical benefits, HWDS is relatively easy for patients to use, often being more cost-effective than polypharmacy and invasive procedures, with minimal to no side effects [16]. While existing HWDS studies have demonstrated consistent short-term pain reduction and increased function, only a few have investigated longer effects. We sought to retrospectively quantify the effects of HWDS in a consecutive cohort of workers’ compensation claimants with longstanding chronic refractory pain, who had already failed non-pharmacologic, pharmacologic, and surgical management. Despite the challenges in offering effective treatment for these very chronic patients, we hypothesized that intervention with HWDS would still have significant, lasting effects in reducing pain and increasing function, thus improving quality of life (QoL).

## 2. Materials and Methods

### 2.1. H-Wave^®^ Stimulation Intensities and Paradigms

The detailed descriptions of stimulation intensities and paradigms can be found in a recent critical review of the H-Wave^®^ pre-clinical and clinical literature [13]. Briefly, at 1000 ohm load, H-Wave^®^ device delivers a current between 0 and 35 mA, a voltage between 0 and 35 V, a pulse duration up to 5 ms, and treatment components of 2 and 60 Hz [13].

### 2.2. Data Source and Study Design

This is a retrospective analysis of data from a consecutive cohort of legacy workers’ compensation claimants treated at the Jeffrey E. Hazelwood MD, PC clinic (Lebanon, TN, USA), who were prescribed HWDS (following a successful 30-day device trial) for refractory chronic pain reported between February 2018 to November 2019. The study time interval was selected to avoid potential issues related to COVID-19 and closure of a remote office. All participants had provided informed consent regarding their health outcomes and no protected health information was reported. This study was approved by the South Texas Orthopaedic Research Institute Institutional Review Board (study approval number: STORI11212021-1 and date of approval: 21 November 2021). General enrollment criteria for this dataset included being aged 18 or older, with well-established post-injury chronic pain having failed multiple prior treatments. All participants received in-clinic personalized instruction by a certified H-Wave^®^ trainer on how to properly apply and operate the device upon initiation of a 30-day trial period. Continuation and purchase of a home device only followed documented trial success, clearly noting improvements in function and perceived pain. Patients were typically followed every 3–6 months for approximately 2 years, completing several widely recognized and validated health questionnaires including the Visual Analog Scale (VAS), Brief Pain Inventory (BPI), Pain Disability Questionnaire (PDQ), Patient Health Depression Questionnaire (PHQ-9), and Generalized Anxiety Disorder (GAD-7) at various intervals during the defined study period. Patients that did not subsequently purchase an H-Wave^®^ device, usually having failed a 30-day trial, or who did not consistently complete post-trial assessments, were excluded from the primary study group.

### 2.3. Data Collection

Pre- and post-trial assessments were administered to patients who elected to continue using an HWDS device following successful trial. Patient demographics (age, gender, race, weight, height, and work status) and medical history (prior surgery and other treatments including TENS, physical therapy, and acupuncture) were documented. Preoperative use of pain medications, either opioid and/or a polypharmacy regimen, was reported. Symptom duration or date of injury (DOI) to trial and body part affected (low back, neck/upper back, leg, shoulder, hip, knee, ankle/foot, chest, and pelvis/groin) were recorded. Patient-reported outcome measures were collected during clinic visits, including VAS, BPI, PDQ, PHQ-9, and GAD-7. Study patients were also directly surveyed by the device manufacturer with a pre-defined set of questions regarding their HWDS experiences, including functional status and pain measures. Data variables were independently compiled into a summary spreadsheet prior to subsequent independent statistical analysis. Based on a combination of collected subjective and objective data, overall patient outcomes were designated as poor, fair, good, or excellent.

### 2.4. Statistical Analysis

The primary outcomes were patient-reported experiences following prolonged HWDS device use. Survey responses were analyzed using contingency analysis for categorical variables, logistic regression analysis for dichotomized variables, ordinal regression analysis for Likert scale variables, and regression analysis for continuous responses. The means comparison tests were employed for pre–post contrasts, using chi-squared and *t*-tests for categorical and continuous variables, respectively. Bivariate analysis assessed the association between survey responses. Stepwise linear regression analysis was performed to assess the correlation between outcome responses and DOI to trial time. In all testing, significance was established when *p*-value < 0.05 or the 95% confidence intervals (CI) for odds ratios excluded 1.0. All statistical analyses were conducted using SAS, version 9.4 (Cary, NC, USA).

## 3. Results

### 3.1. Cohort and Exclusion

**Figure 1:**
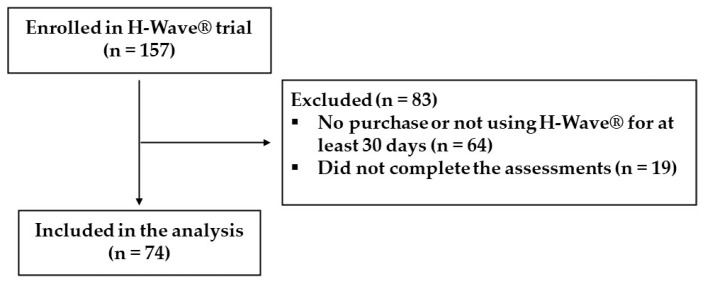
Inclusion/exclusion flow diagram.

Of the 157 total consecutive HWDS patients, 64 participants enrolled in a trial but did not subsequently purchase the H-Wave^®^ device or failed to use it for at least 30 days. A review of 19 trial success patient charts revealed inadequate data for assessing clinical efficacy, with a documentation failure to report the critical outcome measures for pre–post assessments. With the exclusion of those patients (Excluded (*n* = 83)), the final study group consisted of a sample of 74 patients, who successfully completed a 30-day device trial and for which adequate data were collected (Figure 1). Any missing assessment values were removed for each analysis.

### 3.2. Safety

Zero adverse or severe adverse effects were reported throughout the HWDS study duration.

### 3.3. Demographics and Clinical Presentation

The study group demographics and aspects of clinical presentation are recorded in Table 1 and Table 2, including data on age (mean 54.4), gender (nearly equal), ethnicity (82% white), weight, and BMI (mean 206 ± 55 lbs. and 31.5 ± 7.2 Kg/m^2^; mild–moderate obesity [17]), workers’ compensation status (99%), and attorney representation (24%). Low back chronic symptoms predominated (69%), followed by neck (20%), leg (20%), and shoulder (14%) complaints, with many patients reporting pain in multiple body regions. Of those reporting low back pain, only 43% used the device exclusively for it (e.g., radicular and other symptoms). While radiculopathy could not be accurately studied, approximately one-third of low back complaints were associated with lower extremity symptoms suspicious of such. Regarding the previous treatments utilized, 97% of the study group had attended physical therapy, 92% had used a TENS unit, 73% had undergone related surgery, and 9% had tried acupuncture. The average date of injury (DOI) to trial initiation was 7.75 years (95% CI = 6.3–9.2 years), with a mean follow-up of 21.57 ± 12.24 months.

### 3.4. Quality of Life-Related Assessments

Several QoL-related responses are recorded in Table 3**.** Using a Visual Analog Scale (VAS) as one universally reported metric of subjective pain, there was a statistically significant average patient-reported reduction of −2.10 (CL −2.54 to −1.66; *p* < 0.0001), representing an overall 35% improvement. Similarly, comparing pre- and post-treatment Brief Pain Inventory (BPI) scores, a statistically significant improvement (mean 28.2%) was observed (*p* < 0.0001). The study group patients using the Patient Health Depression Questionnaire (PHQ-9) also reported a 24.4% reduction in depression (*p* = 0.0096), as well as 31.0% reduction in anxiety (*p* = 0.0024) using the Generalized Anxiety Disorder (GAD-7) measures.

**Figure 2:**
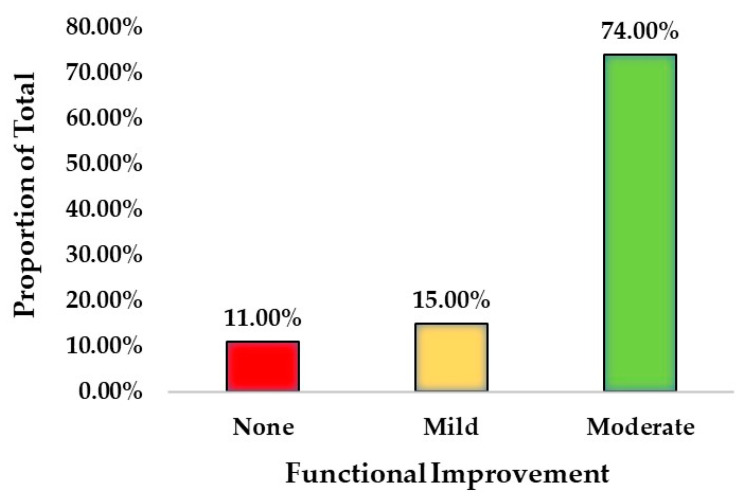
Functional improvement with H-Wave treatment.

In terms of functional status (Table 4 and Figure 2), there was a strong statistical significance (*p* < 0.0001) that the study group patients (89%) were highly likely to experience mild or moderate functional improvement with H-Wave treatments, while 74.0% reported “moderate” or better benefits. The Pain Disability Questionnaire (PDQ) scores also reflected a 35% an overall improvement in disability/function (*p* < 0.0001).

### 3.5. Work- and Medication-Related Findings

The work- and medication-related data are recorded in Table 5 and Table 6 for this workers’ compensation cohort. Among the claimants not working prior to trial, only 6.1% subsequently returned to work; however, of those who were working, 87.5% were able to continue working following HWDS treatment. Regarding opioids, 48.8% of cohort users decreased or stopped usage, while 41.5% completely stopped using narcotics. Similarly, for non-opioid polypharmacy, 36.8% of users decreased their usage, with 24.4% completely stopping the drugs following HWDS treatment.

### 3.6. Overall Outcome/Satisfaction and Effects of Chronicity

**Figure 3:**
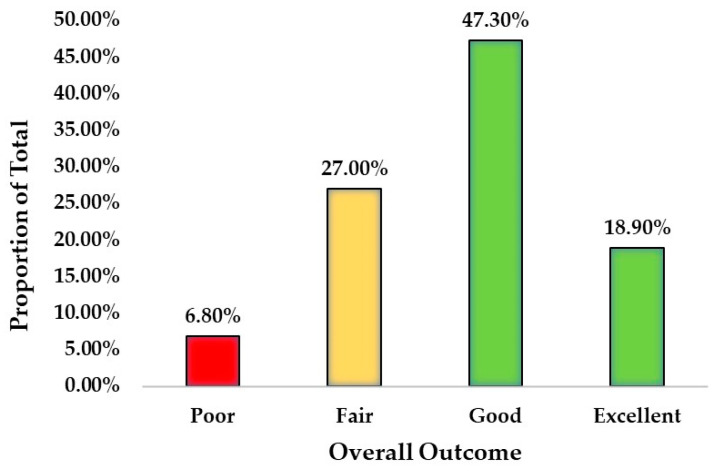
Overall outcome with H-Wave treatment.

The overall outcome/satisfaction results are recorded in Table 7 and Figure 3. Of the 74 HWDS study group patients, it was statistically significant that 66.2% had good to excellent outcomes (*p* = 0.0038).

Table 8 demonstrates that upon limited comparative analysis of the trial failure group (*n* = 63, due to one outlier) with the study group, it was statistically significant that prolongation by a mean of 2.7 years of DOI to trial pain chronicity was more likely to portend trial failure (*p*-value = 0.0225). A logistic analysis of the combined trial failure and study group patients (*n* = 138) also indicated with statistical significance that longer chronicity was associated with poorer outcomes, defined as either trial failure or poor to fair treatment results (*p*-value = 0.0204).

## 4. Discussion

While several previous studies have reported encouraging short-term benefits of H-Wave^®^ device stimulation (examples including significant subjective neuropathic pain reduction and functional gains following rotator cuff repair [18,19]), longer-term outcomes beyond 3-month follow-up have not been well investigated. This HWDS retrospective cohort study, with 2-year mean follow-up, was conducted on a particularly difficult to treat chronic pain population, evaluating improvements in pain, functional status, and psychosocial measures. It is hypothesized that any statistically significant benefits observed in such a refractory “end-stage” cohort might translate to even more promising outcomes and hope for other chronic pain patients who are less challenged by their circumstances.

HWDS treatment reduced subjective pain in 66/73 patients, with 82% of these reporting at least a “moderate” improvement, and a 35% relative VAS score reduction across the entire cohort. This is particularly promising, considering that these patients had already failed to improve with multiple conventional pain treatments. These results were surprisingly comparable to an older study where 78% of patients, with much less chronicity, experienced over 25% pain reduction [19,20]. While this study found several other additional HWDS benefits beyond basic pain reduction (as has been previously reported [13]), a 35% relative increase in functional measures (PDQ score), as well as some improvement in function reported in 89% of the cohort, may be the more profound QoL finding. Of the longer-term psychological benefits, another improved QoL indicator has now also been observed, highlighted by 24% and 31% improvements in depression and anxiety scores, respectively. Such quantification of the psychological benefits of HWDS has not been previously reported, although there has been clear evidence of an association between chronic pain and psychological status [21,22,23].

An important relationship exists between chronic pain and polypharmacy usage, which was defined for the purposes of this study as the use of four or more medications to manage pain including, but not limited to, opioids and other controlled substances, NSAIDS, and acetaminophen [24]. Several studies have shown that polypharmacy use is significantly associated with higher rates of hospitalization (18–28%) and mortality (23–34%) [25,26]. Any reduction in polypharmacy usage, as has been achieved in this study, minimizes the risk of major adverse effects, including tolerance, drug overdose, and death [27]. Polypharmacy issues additionally place financial burdens on patients and payers, highlighting that improvements in chronic pain treatment with non-pharmacologic options will likely result in both physiological and cost benefits [10]. Considering increasing legislation restricting opioid and controlled substance prescriptions, along with the continuing North American opioid crisis, there is a clear imperative to identify safer alternative pain treatment options [28,29]. This study’s finding of decreasing or stopping polypharmacy in 38% of such users represents a step in the right direction, providing further support for the consideration of HWDS to treat chronic pain. More impressively, 49% of the study group patients taking opioids prior to the device trial subsequently reduced or stopped their opioid usage. While previous studies have reported medication reduction over a short-term follow-up, this study has demonstrated longer-term sustained benefits, suggesting that HWDS can effectively address the need to reduce opioid and polypharmacy usage, thereby reducing adverse events and mortality.

While this study demonstrated strong HWDS effects on keeping workers working, the fact that several non-working patients returned to work is remarkable. Workers’ compensation claimants have a reduced financial incentive to return to work, with data from the National Conference of State Legislatures indicating only a 4.9% chance of returning if over two years have passed since the initial absence [30]. This claimant cohort with chronic pain averaging a whopping 7.75 years, had 6.1% of previously non-working individuals returning to work, exceeding the national average [30]. Another recent work-related survey study of first responders who were given access to HWDS at their workplace reported positive experiences in 93%, improved range-of-motion in 93%, pain reduction in 82%, and better job performance in 50%, with some additional sleep and mental health benefits and no reported side-effects [31]. While that group was much healthier with more acute pain issues, the overall positive outcomes were comparable to those of this chronic pain cohort. A previous HWDS meta-analysis reported no adverse effects in over 6000 patients [13,16]. Likewise, this study observed zero adverse effects or complications over a 2-year follow-up period, which is consistent with prior HWDS studies.

This is the first HWDS study to highlight the importance of a standardized 30-day device trial before requesting preauthorization of purchase, something that is of keen interest to payers. Trial failure, without any accrued cost, occurred in 41% of these very chronic pain patients, a figure that would likely be somewhat lower for less chronically affected individuals. It should be noted that participants who failed to report pain and functional benefits during their trial continued to receive other treatments but were not subsequently studied regarding the clinical outcome measures, beyond some basic analyses of demographics, weight, and chronicity. Although there were individual exceptions, chronicity over about 10 years may be more likely to result in trial failure or poorer treatment outcomes. Approximately two-thirds of those who went on to purchase the device after a successful trial significantly benefited from HWDS. These odds of treatment success are quite good, especially considering the lack of other safe and cost-effective alternatives for such end-stage pain patients. Evidence-based medical treatment guidelines, particularly for workers’ compensation, have also suggested completion of an HWDS 30-day trial and prior failure of physical therapy and TENS [32]. From a utilization review perspective, full avoidance of payment for standard 30-day trial failures and long-term minimal device maintenance expense, along with potential for claim resolution, makes the coverage for HWDS particularly attractive.

Despite promising findings and longer-term follow-up in this moderately sized cohort of consecutive chronic pain patients, this study was limited by its retrospective nature and having no comparative or control group. The final study group was significantly reduced in size through disqualification upon trial failure in 64 patients, but also due to inadequate data collection in 19 participants. Reporter bias and other confusion factors may also be an issue, since the collected data were documented by several assisting medical staff or self-reported by patients. Another minor study limitation includes limited applicability to the general population, particularly with skewed race and weight distributions. Future HWDS studies should include double-blinded, randomized control trials to assess improvements more effectively in pain, function, and opioid/polypharmacy use, also focusing on more normalized, non-claimant, and general populations, where better outcomes might be expected.

## 5. Conclusions

H-Wave^®^ device stimulation has demonstrated efficacy in reducing chronic pain and improving function and quality of life, even in a difficult to treat cohort of workers’ compensation claimants with symptom duration averaging almost 8 years. With longer-term follow-up than previous studies reporting clinically significant outcomes, HWDS safely and cost-effectively brought relief to two-thirds of the refractory chronic pain patients completing a successful 30-day device trial, resulting in significant improvement in pain and functional status measures, as well as reduction or cessation (48.8%/41.5%) of opioid use. While mean reduction in self-reported pain decreased by 35%, with similar anxiety and depression benefits, 89% of the study cohort experienced functional improvements. HWDS should be considered a viable non-opioid treatment option for refractory chronic pain patients.

## Figures and Tables

**Table 1:** Demographics of HWDS intervention cohort.

Characteristic	Proportion
Gender	
Male	48.7%
Female	51.3%
Ethnicity	
White	82.4%
Black	14.9%
Hispanic	1.4%
Asian	1.4%
Weight (lbs.)	Mean: 206 ± 55
BMI (kg/m^2^)	Mean: 31.5 ± 7.2
Age (years)	Mean: 54.4 ± 10.6
DOI to trial (months)	Mean: 93.0 ± 73.4
Workers’ compensation	98.7%
Attorney involved	24.3%

**Table 2:** Clinical presentation of HWDS intervention cohort.

Painful Body Part	Proportion (%)
Low back	68.9
Neck/Upper back	20.3
Leg	20.3
Shoulder	13.5
Hip	4.1
Knee	4.1
Ankle/Foot	4.1
Pelvis/Groin	2.8
Chest	2.7

**Table 3:** Mean QoL-related assessment responses.

	Quality of Life Patient-Reported Outcome Measures			
	Pre	Post	Difference	*p*-Value
**VAS**	**6.3 ± 1.7**	4.2 ± 1.8	−2.1 ± 1.9	<0.0001 *
**BPI**	45.1 ± 14.6	32.9 ± 17.3	−12.7 ± 15.8	<0.0001 *
**PDQ**	96.2 ± 28.0	63.0 ± 37.2	−33.5 ± 27.7	<0.0001 *
**PHQ-9**	9.7 ± 6.7	7.3 ± 6.2	−2.4 ± 6.5	0.0096 *
**GAD-7**	7.9 ± 6.2	6.3 ± 5.5	−2.5 ± 5.0	0.0024 *

* Statistically significant.

**Table 4:** Mean function-related assessment responses.

	Functional Improvement	
	Count	Proportion of Total
**None**	8	11.0%
**Mild**	11	15.0%
**Moderate**	54	74.0%

**Table 5:** Bivariate analysis of work-related outcomes.

Count	Returned to Work		
	No	Yes	Total
**Not Working Before Trial**	46 (63.0%)	3 (4.1%)	49 (67.1%)
**Working Before Trial**	3 (4.1%)	21 (28.8%)	24 (32.9%)
**Total**	49 (67.1%)	24 (32.9%)	73 (100%)

**Table 6:** Bivariate analysis of medication-related outcomes.

Count	Post-Trial Opioid Use				
	Stopped After Trial	Reduced After Trial	No Change	Increased After Trial	Total
**No Opioid Before Trial**	NA	NA	30 (41.1%)	2 (2.7%)	32 (43.8%)
**On Opioid Before Trial**	17 (23.3%)	3 (4.1%)	18 (24.7%)	3 (4.1%)	41 (56.2%)
**Total**	17 (23.3%)	3 (4.1%)	48 (65.8%)	5 (6.9%)	73 (100%)
**Count**	**Post-Trial Polypharmacy Use**				
	**Stopped After Trial**	**Reduced After Trial**	**No Change**	**Increased After Trial**	**Total**
**None Before Trial**	NA	NA	21 (28.8%)	7 (9.6%)	28 (38.4%)
**On Drugs Before Trial**	11 (15.1%)	6 (8.2%)	22 (30.1%)	6 (8.2%)	45 (61.6%)
**Total**	11 (15.1%)	6 (8.2%)	43 (58.9%)	13 (17.8%)	73 (100%)

**Table 7:** Mean overall outcome/satisfaction.

	Overall Outcome	
	Count	Proportion of Total
**Poor**	5	6.8%
**Fair**	20	27.0%
**Good**	35	47.3%
**Excellent**	14	18.9%

**Table 8:** Time duration of DOI to trial (chronicity).

Time Between (Months)	Study Group	Trial Failure
**Average ± S.D.**	92.96 ± 73.41	124.51 ± 83.03
**95% Interval**	(75.95, 109.97)	(103.60, 145.42)
**Sample Size**	74	* 63

* Single extreme outlier removed.

## Data Availability

Data are contained within the article.

## References

[1] Chronic Pain and High-Impact Chronic Pain among U.S. Adults, 2019. https://www.cdc.gov/nchs/products/databriefs/db390.htm#:~:text=Interview%20Survey%2C%202019.-,Summary,65%20and%20over%20(30.8%25).

[2] Horppu R., Väänänen A., Kausto J. (2022). Evaluation of a guidelines implementation intervention to reduce work disability and sick leaves related to chronic musculoskeletal pain: A theory-informed qualitative study in occupational health care. BMC Musculoskelet. Disord..

[3] Smith T.J., Hillner B.E. (2019). The Cost of Pain. JAMA Netw. Open.

[4] Pharmacologic Management of Chronic Non-Cancer Pain in Adults. https://www-uptodate-com.proxy.ulib.uits.iu.edu/contents/pharmacologic-management-of-chronic-non-cancer-pain-in-adults?search=Management%20of%20chronic%20pain&topicRef=126111&source=see_link#H1470711919.

[5] Daoust R., Paquet J., Cournoyer A., Piette É., Morris J., Lessard J., Castonguay V., Lavigne G., Huard V., Chauny J.M. (2021). Opioid and non-opioid pain relief after an emergency department acute pain visit. CJEM..

[6] Owusu Obeng A., Hamadeh I., Smith M. (2017). Review of Opioid Pharmacogenetics and Considerations for Pain Management. Pharmacotherapy.

[7] Khademi H., Kamangar F., Brennan P., Malekzadeh R. (2016). Opioid Therapy and its Side Effects: A Review. Arch. Iran. Med..

[8] Miller J., MacDermid J.C., Walton D.M., Richardson J. (2020). Chronic Pain Self-Management Support With Pain Science Education and Exercise (COMMENCE) for People With Chronic Pain and Multiple Comorbidities: A Randomized Controlled Trial. Arch. Phys. Med. Rehabil..

[9] Kaye A.D., Jones M.R., Kaye A.M., Ripoll J.G., Galan V., Beakley B.D., Calixto F., Bolden J.L., Urman R.D., Manchikanti L. (2017). Prescription Opioid Abuse in Chronic Pain: An Updated Review of Opioid Abuse Predictors and Strategies to Curb Opioid Abuse: Part 1. Pain Physician.

[10] Swedish Council on Health Technology Assessment (2006). Methods of Treating Chronic Pain: A Systematic Review.

[11] Ekholm O., Diasso P.D.K., Davidsen M., Kurita G.P., Sjøgren P. (2022). Increasing prevalence of chronic non-cancer pain in Denmark from 2000 to 2017: A population-based survey. Eur. J. Pain.

[12] Eklund K., Stålnacke B.M., Stenberg G., Enthoven P., Gerdle B., Sahlén K.G. (2020). A cost-utility analysis of multimodal pain rehabilitation in primary healthcare. Scand. J. Pain.

[13] Williamson T.K., Rodriguez H.C., Gonzaba A., Poddar N., Norwood S.M., Gupta A. (2021). H-Wave^®^ Device Stimulation: A Critical Review. J. Pers. Med..

[14] Blum K., Ho C.K., Chen A.L., Fulton M., Fulton B., Westcott W.L., Reinl G., Braverman E.R., Dinubile N., Chen T.J. (2008). The H-Wave((R)) Device Induces NODependent Augmented Microcirculation and Angiogenesis, Providing Both Analgesia and Tissue Healing in Sports Injuries. Physician Sportsmed..

[15] Thiese M.S., Hughes M., Biggs J. (2013). Electrical stimulation for chronic non-specific low back pain in a working-age population: A 12-week double blinded randomized controlled trial. BMC Musculoskelet. Disord..

[16] Blum K., Chen A.L., Chen T.J., Prihoda T.J., Schoolfield J., DiNubile N., Waite R.L., Arcuri V., Kerner M., Braverman E.R. (2008). The H-Wave device is an effective and safe non-pharmacological analgesic for chronic pain: A meta-analysis. Adv. Ther..

[17] Apovian C.M. (2016). Obesity: Definition, comorbidities, causes, and burden. Am. J. Manag. Care.

[18] Blum K., DiNubile N.A., Chen T.J.H., Waite R.L., Schoolfield J., Martinez-Pons M., Callahan M.F., Smith T.L., Mengucci J., Blum S.H. (2006). H-Wave, a nonpharmacologic alternative for the treatment of patients with chronic soft tissue inflammation and neuropathic pain: A preliminary statistical outcome study. Adv. Ther..

[19] Blum K., Chen A.L.C., Chen T.J.H., Waite R.L., Downs B.W., Braverman E.R., Kerner M.M., Savarimuthu S.M., DiNubile N. (2009). Repetitive H-Wave device stimulation and program induces significant increases in the range of motion of post operative rotator cuff reconstruction in a double-blinded randomized placebo controlled human study. BMC Musculoskelet. Disord..

[20] Blum K., DiNubile N.A., Chen T.J.H., Waite R.L., Schoolfield J., Martinez-Pons M., Callahan M.F., Smith T.L., Mengucci J., Blum S.H. (2006). The H-Wave small muscle fiber stimulator, a nonpharmacologic alternative for the treatment of chronic soft-tissue injury and neuropathic pain: An extended population observational study. Adv. Ther..

[21] Hooten W.M. (2016). Chronic Pain and Mental Health Disorders: Shared Neural Mechanisms, Epidemiology, and Treatment. Mayo Clin. Proc..

[22] Dueñas M., Ojeda B., Salazar A., Mico J.A., Failde I. (2016). A review of chronic pain impact on patients, their social environment and the health care system. J. Pain Res..

[23] Vadivelu N., Kai A.M., Kodumudi G., Babayan K., Fontes M., Burg M.M. (2017). Pain and Psychology—A Reciprocal Relationship. Ochsner J..

[24] Masnoon N., Shakib S., Kalisch-Ellett L., Caughey G.E. (2017). What is polypharmacy? A systematic review of definitions. BMC Geriatr..

[25] Chang T.I., Park H., Kim D.W., Jeon E.K., Rhee C.M., Kalantar-Zadeh K., Kang E.W., Kang S.W., Han S.H. (2020). Polypharmacy, hospitalization, and mortality risk: A nationwide cohort study. Sci. Rep..

[26] Li Y., Zhang X., Yang L., Yang Y., Qiao G., Lu C., Liu K. (2022). Association between polypharmacy and mortality in the older adults: A systematic review and meta-analysis. Arch. Gerontol. Geriatr..

[27] Benyamin R., Trescot A.M., Datta S., Buenaventura R., Adlaka R., Sehgal N., Glaser S.E., Vallejo R. (2008). Opioid complications and side effects. Pain Physician..

[28] Smith T.L., Blum K., Callahan M.F., DiNubile N.A., Chen T.J., Waite R.L. (2009). H-Wave induces arteriolar vasodilation in rat striated muscle via nitric oxide-mediated mechanisms. J. Orthop. Res..

[29] Overdose Death Rates. https://nida.nih.gov/research-topics/trends-statistics/overdose-death-rates#:~:text=Drug%20overdose%20deaths%20involving%20prescription,increase%20to%2016%2C416%20in%202020.

[30] Workers’ Compensation: Keeping Injured and Ill Workers in the Workforce. https://www.ncsl.org/research/labor-and-employment/workers-compensation-report.aspx.

[31] Williamson T.K., Rodriguez H.C., Han D., Norwood S.M., Gupta A. (2022). Job-Related Performance and Quality of Life Benefits in First Responders Given Access to H-Wave^®^ Device Stimulation: A Retrospective Cohort Study. J. Pers. Med..

[32] ODG by MCG. https://www.mcg.com/odg/.

